# Research progress of neurovascular units involved in ischemic stroke

**DOI:** 10.1002/ibra.12166

**Published:** 2024-06-03

**Authors:** Yu Yang, Hao Tong, Zhuo‐Fan Ye, Zu‐Cai Xu, Tao Tao

**Affiliations:** ^1^ Department of Neurology Affiliated Hospital of Zunyi Medical University Zunyi China; ^2^ Department of Rehabilitation Medicine Guizhou Provincial People's Hospital Guiyang China

**Keywords:** astrocyte, blood–brain barrier, ischemic stroke, neurovascular unit

## Abstract

Ischemic stroke is the most prevalent cerebrovascular disorder in the clinical setting. It results in associated neurological abnormalities due to a variety of factors, including disruption of cerebral arterial blood flow, hypoxia, and ischemic necrosis of local brain tissues. The neurovascular unit (NVU) is a dynamic structural complex that consists of neurons, glial cells, pericytes, vascular endothelial cells, and the extracellular matrix. Many cells work together to preserve the integrity of the central nervous system (CNS) under physiological conditions. However, following ischemic stroke, NVU homeostasis is disrupted along with the development of tissue ischemia and hypoxia, as well as impaired interactions between various components of the NVU. Collectively, the changes result in increased blood–brain barrier permeability, neuronal dysfunction, and functional destruction of nerve conduction bundles, ultimately leading to the clinical manifestation of neurological deficits including motor, cognitive, and speech impairments that hinder the rehabilitation process. In recent years, with continuously expanding research on ischemic cerebrovascular disease, the role of interconnections between different cells in the NVU in ischemic stroke has received increasing attention. To describe new concepts for the prevention and treatment of ischemic cerebrovascular illnesses, this article reviews the interplay between NVU in the pathogenesis of ischemic stroke.

## Graphical abstract

The brain receives less blood when an ischemic stroke develops, which damages neurons and leads to an increased release of Pro‐inflammatory mediators that activate neurovascular unit (NVU). When the tight junctions are broken and the basement membrane is disrupted by matrix metalloproteinases produced by active endothelial and pericellular cells, harmful chemicals flow through the blood–brain barrier (BBB) pore. Pro‐inflammatory mediators are released by activated astrocytes, further destroying NVU and neurons. Microglia dynamically change into reactive and encircling cerebral arteries, hastening the BBB breakdown. BBB dysfunction is made worse when pericytes separate from blood vessel walls or die, which also affects blood flow control.

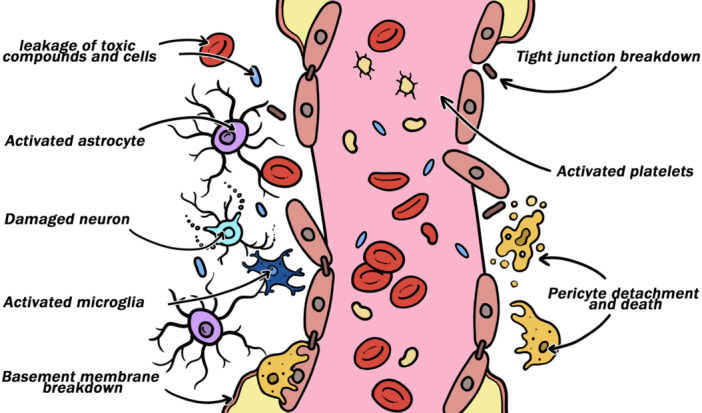

## 1 INTRODUCTION

Ischemic stroke is a neurological disorder caused by insufficient blood supply to the brain due to various reasons. It is the second‐leading cause of death and the primary global cause of disability.^1^ Ischemic stroke accounts for approximately 87% of all stroke cases globally and is becoming increasingly common.^2^ With the implementation of effective reperfusion strategies, intravenous thrombolysis has become the most commonly used treatment for patients with ischemic stroke.^3^ In this regard, tissue plasminogen activators (t‐PA) are widely accepted therapeutic agents. Unfortunately, due to the strict time window of t‐PA treatment, it is only suitable for a limited number of patients.^4^, ^5^ Therefore, further investigation to discover effective and feasible treatment for ischemic stroke is necessary.

**Figure 1:**
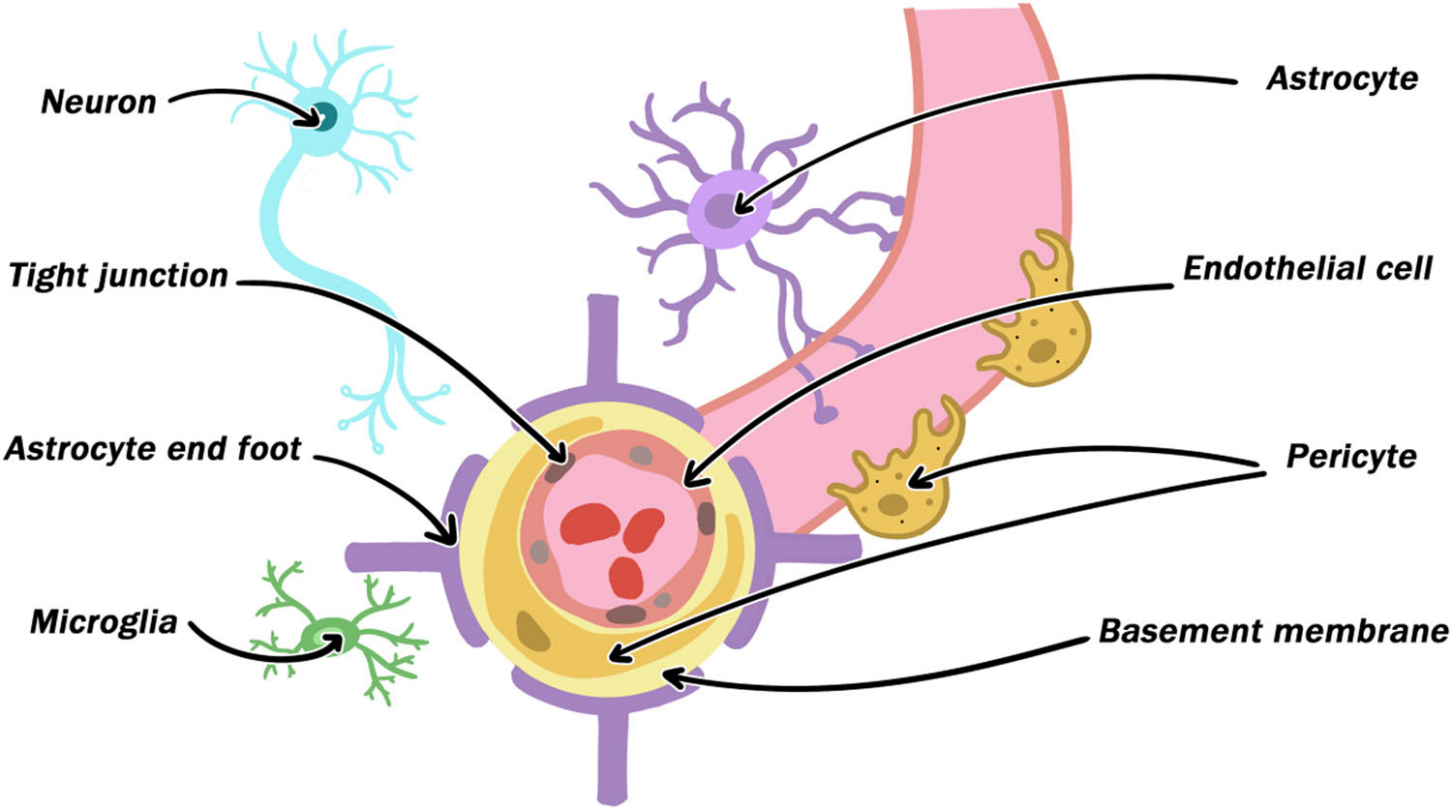
Structural components of the neurovascular unit (NVU). NVU is composed of neurons, astrocytes, microglia, endothelial cells, pericytes, the extracellular matrix, and the basement membrane. The endothelial cells that form blood vessels are enveloped in the basement membrane and bound by tight junction proteins. The pericytes are situated between the terminal feet of the astrocytes and endothelial cells, and the astrocytes are situated in the brain parenchyma, in contact with the pericytes and endothelial cells on the capillary wall. [Color figure can be viewed at wileyonlinelibrary.com]

The neurovascular unit (NVU) is a special cerebrovascular structure composed of vascular cells, glial cells, neurons, and the extracellular matrix, which plays an important role in maintaining brain function (Figure 1).^6^ Several cell types work together to preserve the integrity of the central nervous system (CNS) under physiological circumstances. However, under pathological conditions, the signals of various cell components of the NVU are destroyed, causing more serious damage. On the one hand, the damage to the neurons results in a chain of acute injury reactions.^7^ On the other hand, it facilitates the development of new blood vessels, aids in NVU function restoration, and supports nerve repair.^8^ Therefore, current research paradigms on brain protective therapies for ischemic stroke have shifted from using a single neuron to NVU as a therapeutic target. In this article, advancements in understanding the underlying causes of NVU dysfunction following ischemic stroke are reviewed.

## 2 POSSIBLE MECHANISM OF BLOOD–BRAIN BARRIER IN ISCHEMIC STROKE

An essential part of the brain microcirculation is the blood–brain barrier (BBB), which primarily consists of capillary endothelial cells, pericytes, the basement membrane, and astrocytes end foot.^9^ Under normal conditions, the BBB expresses a variety of ion transport proteins, and ion channels provide essential nutrients for the brain tissue and eliminate metabolic waste from the brain, thereby effectively maintaining a stable state of the CNS.^10^ The BBB acts as a highly selective barrier and prevents certain substances from passing through it. To achieve this, the BBB relies on junction adhesion molecules and tight junction (TJ) proteins to tighten the connection between astrocytes and endothelial cells as well as pericytes, fully ensuring the integrity and stability of the BBB structure.^9^, ^11^ By isolating the CNS from the peripheral blood circulation, as described above, the BBB successfully restricts and controls the movement of chemicals, ions, and cells between the blood and CNS; this makes it difficult for most harmful components, such as molecules, viruses, and inflammatory factors, to pass through the BBB, so as to maintain the normal physiological function of the CNS.^10^, ^11^, ^12^ However, as part of the pathological process of ischemic stroke, neuroinflammatory reactions mediated by glial cells, pro‐inflammatory cytokines, and matrix metalloproteinases (MMPs) can lead to structural dysfunction of the BBB, which can exacerbate ischemic brain damage.^13^, ^14^ However, during the chronic stage of ischemic stroke, the aforementioned inflammatory mediators promote neuronal survival, axon growth, and NVU remodeling, thereby playing an important role in nerve repair.^15^, ^16^

### 2.1 Endothelial cells

Vascular endothelial cells prevent cells and molecules from passively entering the brain through tight intercellular connections, forming a physical barrier as part of the BBB.^17^ Occludins, claudins, and connective adhesion molecules belong to TJ proteins. Among them, claudin‐5 is highly expressed in endothelial cells and has the closest connection, which limits the pore size of the BBB to below 800 Da.^18^ As the first line of defense for NVU, endothelial cells and the tight connections between them maintain the permeability of the barrier, limiting the entry of dangerous substances into the brain, such as poisons. The currently accepted theory is that the BBB is not only a physical “barrier” but also a metabolic interface that is dynamic.^19^ The interaction between NVU partners and the integrity of the BBB is key to brain homeostasis. By monitoring changes in the shear stress of the endothelial wall and the supply of oxygen and vital nutrients to the brain tissue, the interaction between capillary and neuronal components of neurovascular coupling controls cerebral blood flow.^20^ The breakdown of the BBB causes toxic molecules to flood into the brain, causing neuronal damage and neurodegenerative changes.^21^

After an ischemic stroke, endothelial cells undergo cytoskeletal rearrangement, increased cell transport, and changes in TJ proteins, leading to progressive BBB dysfunction. Oxidative stress and inflammation can cause endothelial cell damage and irreversible endothelial injury.^22^ In the meantime, endothelial cells can also produce MMPs under the stimulation of pro‐inflammatory cytokines and free radicals, forming TJs and degrading the extracellular matrix. Endothelin‐1, another molecule produced and released by endothelial cells, regulates thrombosis by activating platelets, reducing local cerebral blood flow, and causing cerebral infarction.^23^ In the subacute phase, autophagic bodies are observed in the endothelial cells of the ischemic hemisphere.^24^ Whether accelerated autophagy is beneficial for survival is unclear; however, recent studies have demonstrated that endothelial autophagy plays a beneficial role in improving BBB breakdown and TJ loss after ischemia.^11^

### 2.2 Basement membrane

The basement membrane, located on the abdominal side of endothelial cells or on the basal side of epithelial cells, is an amorphous structure. Previous studies on the BBB focused mainly on its cellular components, but some studies showed that noncellular components of the basement membrane also actively participate in the regulation of vascular integrity.^25^ The basement membrane is composed of extracellular matrix proteins such as laminin and collagen type IV secreted by pericytes, vascular endothelial cells, and astrocytes. Laminin‐411 and ‐511 are produced by endothelial cells, whereas laminin‐111 and ‐211 are produced by astrocytes. The expression of laminin helps vascular smooth muscle cells to differentiate into contractile phenotypes and keeps pericytes in a resting state, maintaining the normal function of the BBB.^26^

After an ischemic stroke, activated endothelial cells and pericytes produce MMP, which degrades the basement membrane.^27^, ^28^ At the same time, hypoxia and vascular fibrosis can also activate microglial cells to release proteases and free radicals, attack TJ proteins, and destroy the basement membrane and myelin sheath, leading to demyelination in the brain's white matter.^29^ When a lesion involves capillaries, owing to their lack of smooth muscles, amyloid proteins can be directly deposited in the basement membrane, causing increased vascular fragility and luminal stenosis, leading to pathological changes such as ischemia and microbleeding.^30^

### 2.3 Pericytes

The formation and integrity of the BBB, angiogenesis, and toxin removal are critical functions of pericytes.^31^ The protrusions of pericytes wrap around the entire length of the capillaries and are enmeshed in the basement membrane of the endothelial cells, forming tight contact with endothelial cells through gap and adhesive interactions.^32^ In the physiological state, transforming growth factor β (TGF‐β) expressed in the precursor form of pericytes causes the pericytes to differentiate and mature. Angiopoietin‐1 and ‐2 as well as vascular endothelial growth factor (VEGF) secreted by pericytes promote endothelial cell proliferation. Vascular remodeling, stability, and maturation are caused by the interaction between platelet‐derived growth factors released by endothelial cells and the platelet‐derived growth factor receptors, which are secreted by pericytes.^33^ Pericytes promote the emergence and growth of new capillaries during the initial stages of microvascular development. Pericytes support endothelial cell differentiation and decrease endothelial cell proliferation at later stages, promoting blood vessel development. In addition, pericytes promote basement membrane formation by secreting type IV collagen, mucopolysaccharides, laminin, and other substances. This helps to normalize the BBB and regulates its permeability after the onset of stroke.^34^

Pericytes play a critical role in regulating blood flow after stroke and maintaining vascular stability by secreting basement membrane proteins. They are able to control capillary diameter through calcium signaling and inhibit the expression of genes that promote vascular permeability.^35^ Pericytes are observed to migrate from cerebral microvessels during the acute phase of ischemic stroke, which eventually leads to loss of pericyte cover due to their separation from the microvessel wall and cell death.^36^ The rapid activation of MMP‐9 secreted from pericellular cell bodies was thought to have caused the underlying TJ complex to be degraded in a later study of photothrombotic stroke in mice, which resulted in plasma leakage between pericellular cell bodies and nearby capillary walls. The combination of endothelial cell response processes to stroke implies that pericytes may use MMP‐9 to actively migrate from endothelial cells to affected areas and participate in vascular remodeling and repair of NVU after stroke.^32^

Studies have shown that sustained hypoxic‐ischemic injury can lead to oxidative stress and pericellular contraction, eventually resulting in cell death.^37^ In addition to affecting blood flow regulation and BBB function, pericyte loss also has an impact on neuronal survival due to reduced levels of pleiotropic proteins secreted by pericytes.^38^ At the same time, signal transduction and transcriptional activator 3 are activated in cells during ischemia and hypoxia before hypoxia‐inducing factor 1α response, contributing to angiogenic neurogenesis and thus promoting neural recovery.^39^ To prevent BBB failure, promote angiogenesis, and stabilize ischemic stroke, it may be beneficial to enhance the interactions between pericytes and endothelial cells by controlling the aforementioned signaling pathways.

## 3 POSSIBLE MECHANISMS OF NEURONS IN ISCHEMIC STROKE

Neurons are a core component of the brain, and the ability of adult neurons to proliferate or regenerate is limited.^40^ Numerous studies have shown that neuronal death is a significant contributor to the poor prognosis of ischemic stroke.^41^ Interruption of the neuronal blood supply caused by ischemia subsequently facilitates the pathophysiological cascade of excitotoxicity, free radical release, protein misfolding, mitochondrial death pathways, apoptosis, necrosis, autophagy, and inflammation. The extent of neuronal loss in the brain regions affected by ischemic stroke heavily influences the severity and cause of death.^42^

Excitatory toxicity is the primary cause of neuronal injury following cerebral ischemia. Glutamate (Glu) is one of the most important excitatory neurotransmitters, and ischemia stimulates the release of Glu in large quantities. Glutamate transporters, such as glutamate transporter 1 and cystine/glutamate reverse transporters, control the concentration of Glu in the brain, and neurons do not maintain homeostatic levels of Glu.^43^ Excess Glu activates multiple cellular pathways including Ca^2+^ influx, oxidative stress, protease activity, and endonuclease breakdown, which are the primary causes of delayed neuronal degeneration. In these processes, adenosine 5′ triphosphate (ATP) is consumed, the mitochondrial membrane potential is lost, respiratory chain dysfunction occurs, and activation of the mitochondrial death pathway finally results.^44^ Reactive oxygen species (ROS) are mainly produced by astrocytes and microglia in the CNS. Reduced nicotinamide adenine dinucleotide phosphate (NADPH) oxidase, which produces most of the superoxide anion, is the main driver of oxidative stress in cerebral ischemia. By inhibiting the expression of the S100β gene, the level of superoxide dismutase scavenging oxygen free radicals increases and the level of malondialdehyde decreases, which may reduce the number of apoptotic neurons.^45^

Additionally, with the advent of the NVU concept, neurons are no longer studied in isolation. In the coculture system of neurons, astrocytes and microvascular endothelial cells treated with catalpol and puerarin lyophilized powder, it was found that protection of the entire NVU can expedite the recovery of nerve function by decreasing levels of inflammatory factors such as nuclear factor kappa B (NF‐κB)/p65, tumor necrosis factor‐α (TNF‐α), interleukin (IL)‐1β, and IL‐6 and increasing levels of protective factors such as erythropoietin, erythropoietin receptor, VEGF, and growth‐related protein 43.^46^

## 4 POSSIBLE MECHANISM OF GLIAL CELLS IN ISCHEMIC STROKE

### 4.1 Oligodendrocyte

In the NVU, oligodendrocytes play a key role in myelin production, an insulating myelin sheath structure that aids the effective jump transmission of bioelectrical signals, preserving as well as defending normal neuronal function. Impaired nerve function results from the loss of oligodendrocytes.^47^ Several other neurotrophic and growth factors, including glia‐derived neurotrophic factor (GDNF) and insulin‐like growth factor 1 (IGF‐1), are secreted by oligodendrocytes and promote neuronal survival as well as axonal growth.^48^ Endothelial cells promote the formation of oligodendrocyte progenitor cells by secreting trophic substances such as brain‐derived neurotrophic factor (BDNF), basic fibroblast growth factor (bFGF), and VEGF‐A, to promote oligodendrocyte migration. Following ischemia, oligodendrocytes release MMP‐9, causing early breakdown of the BBB.^49^

Immunofluorescent labeling of oligodendrocytes and their myelin‐associated proteins, oligodendrocyte‐specific proteins, and myelin basic proteins has shown that oligodendrocyte‐specific proteins and myelin basic proteins appear simultaneously in the early stages of ischemia. The myelin basic protein response is significantly increased in the ischemic center, clustered near the damaged blood vessels, and gradually decreased in the striatum, ischemic marginal region, and lateral neocortex.^50^

### 4.2 Astrocytes

Glial cells have crucial roles in maintaining brain homeostasis and establishing the BBB among which astrocytes may be the most prevalent cell type in the CNS.^51^ In both healthy and damaged brains, astrocytes serve important roles. As an important component of the BBB, they extend from the cell body to nonoverlapping astrocytes that form glial cell boundaries when wrapping blood vessels or meningeal‐related parenchymal substrates at the distal end, thereby maintaining the integrity of the BBB and forming a secondary barrier that further limits the entry of peripheral immune cells into the CNS.^52^, ^53^ In the brain, astrocytes release a variety of molecular mediators, such as prostaglandins, nitric oxide (NO), and arachidonic acid, which can also regulate blood flow by detecting the level of Glu in synapses and regulating the concentration of Ca^2+^ in their terminal pods, thereby releasing glial transmitters.^54^ Therefore, astrocytes are essential for regulating and maintaining BBB function.

Astrocytes have many foot processes that fill the gap between the neuronal cell body and process, keeping neuronal structure stable and acting as a separator. They secrete neurotrophic factors that support neuronal growth, development, survival, and function, as well as provide energy metabolism and participate in various metabolic pathways. In response to brain injury, astrocytes can undergo gliosis, forming scar tissues.^55^ After a stroke, a significant proportion of astrocytes continue to function and contribute to neuroprotection. Therefore, astrocytes have emerged as potential targets for stroke treatments.

#### 4.2.1 Reactive astrogliosis

After an ischemic stroke, astrocytes undergo significant morphological changes, often referred to as reactive astrocyte hyperplasia. Reactive astrocytes are now commonly understood to be “the participation of astrocytes in molecularly defined programs involving changes in transcriptional regulation, as well as biochemical, morphological, metabolic, and physiological remodeling, ultimately leading to the acquisition of new functions or the loss or upregulation of homeostasis function in response to pathological conditions”, according to a recent review.^56^ After cerebral ischemia, astrocytes undergo a transformation from their typical thick form into supernutrition stellate, which is followed by a protracted period of ischemic expansion.^53^ During the acute phase (1–4 days after ischemia), reactive astrocyte proliferation is increased, marked by upregulation of GFAP. High‐resolution imaging also showed a continuous increase in the protrusions of polarized astrocytes from the subacute phase (4–8 days after ischemia) and progressive formation of mature glial scars until the chronic phase (8–14 days after ischemia).^57^

The acute increase of Ca^2+^ signaling in astrocytes and the subsequent release of Glu and gamma‐aminobutyric acid (GABA) may be the initial steps after ischemia. Many downstream signaling intermediaries, including the phosphatase calcineurin, which activates the NFAT or N‐cadherin pathway, can be controlled by Ca^2+^.^58^ Transcriptional regulators or reverse transcription inhibitors of various pathways (such as SOCS3 for the JAK‐STAT3 pathway and IB for the NF‐κB pathway) are also involved in triggering the production of GFAP. These pathways include signal transducers and activators of transcription 3 (STAT3), p38 MAPK, NF‐κB, and transforming growth factor (TGF) signaling pathways.^59^ Reactive astrogliosis is traditionally considered to be responsible for the formation of glial scars. However, there is growing evidence that reactive astrocytes can also perform beneficial functions. Understanding the precise transformation of astrocyte responses to stroke is crucial for developing therapeutic interventions.

#### 4.2.2 Interactions between reactive astrocytes and other cells

Studies have shown that neuroinflammation and cerebral ischemia are mediated by the secretion of IL‐1α, TNF‐α, and complement 1q (C1q) by classically activated microglia (M1 type). C1q induces astrocyte polarization in the neurotoxic A1 phenotype.^60^ Reactive astrocytes lose their capacity to support neuronal survival, development, synapse formation, and phagocytosis when converted into pro‐inflammatory phenotypes^61^; they can also release a variety of classic complement cascade components, increase synaptic degeneration, and induce neuronal and oligodendrocyte death.^62^ Astrocytes during cerebral ischemia mainly activate NF‐κB, signal transducers, and the STAT3 pathway to induce inflammatory cytokines (IL‐6, IL‐1α, IL‐1β, TNF‐α, and interferon‐γ, among others) and release neurotoxic mediators (including NO and ROS), leading to neuronal damage and death.^63^

In contrast to the A1‐type astrocytes, polarizing astrocytes of the A2 phenotype also perform phagocytosis and promote synaptic formation as well as neuron survival.^64^ The characteristic markers of type A2 astrocytes include n‐pentametin, S100, calcium‐binding protein A10, and sphingosine‐1‐phosphate receptor 3.^65^ On the one hand, type A2 astrocytes can produce erythropoietin, nerve growth factors (NGFs), IGF‐1, CNTF, bFGF, BDNF, and GDNF to protect neurons following cerebral ischemia.^66^ In contrast, A2‐type astrocytes can activate regulatory proteins such as STAT3, ras homologous family member A, hypoxia‐inducible factor 1 subunit, and EPO, which are involved in neuroinflammation and nerve repair.^67^, ^68^ According to in vitro studies, activated astrocytes release IL‐17A via the MAPK pathway, which can boost the neural differentiation ratio of neural stem cells and encourage their differentiation into neurons.^36^ After primary human astrocytes overexpress interferon regulatory factors, a shift from the pro‐inflammatory phenotype A1 to the anti‐inflammatory phenotype A2, which is associated with neuroprotection, has been observed.^69^ In vitro primary astrocyte cultures and in vivo experiments in mice have shown that the overexpression of prokinetic keratin induces the expression of key antioxidant genes and A2 reactive markers of the A2 astrocyte phenotype, playing anti‐inflammatory and antioxidant roles.^70^

When ischemic stroke occurs, the reduced lactate supply of astrocytes can promote stroke‐induced neurodegeneration.^71^ Astrocytes are also able to provide neuroprotection and neuronal recovery by transferring their own endogenous mitochondria to neurons.^72^ It is well known that reactive astrocytes are a significant source of steroids, particularly estrogen. Following brain ischemia, the expression of the enzyme aromatase and its production of 17‐estradiol in astrocytes are upregulated. When estrogen was downregulated in astrocytes of mice with aromatase knockouts, there was less astrogliosis and more neuronal damage under ischemic conditions.^73^ It is interesting to note that in the ischemic brain, neurons also secrete estrogen 17‐estradiol, which is essential for astrocyte activation and the release of neurotrophic factors.^74^ Recent studies have demonstrated that exosomes produced by astrocytes can increase the survival of neuronal cells in ischemic conditions.^75^ Astrocyte‐derived exosomes can regulate neuronal apoptosis by mediating miR‐92b‐3p and miR‐190b.^76^, ^77^ Exosomal miR‐361 from astrocytes inhibits the AMPK/mTOR signaling pathway by targeting cathepsin B (CTSB), having both in vitro and in vivo neuroprotective effects.^78^

Brain edema and hemorrhagic transformation are serious side effects of acute ischemic stroke caused by BBB breakdown. Astrocytes have a significant impact on cytotoxic edema, especially when it comes to the water channel aquaporin‐4 (AQP‐4).^79^ A large amount of AQP‐4 exists on the surface of the soft meninges of astrocytes and on the terminal foot membrane of surrounding blood vessels.^80^ AQP‐4 has the function of regulating fluid flow in the interstitial space and maintaining the interstitial environment required for normal neuronal function.^53^ Following a stroke, astrocyte‐secreted MMPs and VEGF also boost blood vessel permeability and vasogenic edema.^81^ To preserve the integrity of the BBB, neutralizing IL‐9 may cause astrocyte‐derived VEFG‐A to be downregulated.^82^ However, reactive astrocyte‐derived pentraxin‐3 might protect the integrity of the BBB by controlling VEGF‐related mechanisms in the areas around infarcts, which could be a protective mechanism.^83^ Pericytes, another crucial element of the BBB, have receptors for many vasoactive signaling molecules. Pericyte differentiation is controlled by astrocyte laminin, which also preserves BBB integrity.^84^

### 4.3 Microglia

Microglia, derived from bone marrow monocytes and hematopoietic stem cells, are primarily located in the gray matter near neuronal cell bodies or around small blood vessels.^85^, ^86^ They play crucial roles in various physiological processes during embryonic development,^87^ including regulating the growth, development, and number of neurons, participating in synaptic pruning, shaping neural circuits, supporting other CNS cells, and promoting vasculature development.^88^ As the resident immune cells of the CNS, microglia are the first responders to CNS damage. After an ischemic stroke, microglia sense changes in the internal environment through the toll‐like receptors (TLRs) on their surfaces and are rapidly polarized into different phenotypes.^89^

#### 4.3.1 Reactive microglia

After cerebral ischemia, microglia rapidly polarize into M1 microglia and reduce the survival rate of neurons after stroke by secreting various active substances and participating in the pathogenesis of ischemic stroke.^90^ Microglia activate and release a variety of cytokines that promote inflammation (including TNF‐α, IL‐1β, IL‐6, and IL‐8), which collectively lead to dysfunction of neuronal survival, proliferation, differentiation, nonspecific injury to cell function, inhibition of neuronal regeneration and repair, and aggravation of inflammation. Damage to the brain tissue is involved in neuronal degeneration and damage to the cerebrovascular barrier,^91^ which further causes degeneration and necrosis of neurons. Moreover, overactivated M1‐type microglial cells release a large number of cytotoxic substances, such as ROS, NO, glutamic acid, and peroxide, which amplify the inflammatory cascade reaction, aggravate neuronal damage, and damage adjacent neurons, resulting in neural network dysfunction. In addition, the inflammatory cascade can block the microcirculation and affect blood supply in the ischemic penumbra.^92^

IL‐4 is crucial for the polarization of microglial cells and recovery of neurons after an ischemic stroke. After an ischemic stroke, the secretion of IL‐4 by ischemic neurons increases significantly, which promotes the polarization of microglial cells in the ischemic penumbra to the M2 type as well as phagocytosis of injured cells, and can induce the regeneration of injured nerve tissue and formation of blood vessels in the injured area.^93^ Activated microglial cells at the injured site and its periphery express many neurotrophic factors that promote neuronal regeneration.^94^ After an ischemic stroke, BDNF released by M2‐type microglial cells can increase synaptic plasticity, promote neuronal axonal extension and branching, increase the density of neuronal dendrites and dendritic spines, and promote the formation of new neural circuits.^95^ At the same time, some neurotrophic factors such as NGF can regulate the immune response of microglial cells. For example, NGF can reduce neuroinflammation and promote neuronal regeneration by regulating the surface molecules of microglial cells, especially by reducing the levels of major histocompatibility complex II.^96^

The temporal and spatial dynamics of different microglial phenotypes are critical following ischemic stroke.^97^ At the beginning of ischemia, M1‐type microglia are activated, showing amoebic‐like shape changes in the ischemic central region and branching in the penumbral region, with thicker and shorter protuberations than M1‐type microglia in the inactivated state. M2‐type microglial cells increase rapidly within 24 h of transient injury, and their phenotypic markers are highly expressed in the ischemic center.^98^ At this time, microglial cells play a crucial role in reducing nerve cell injury, enhancing nerve regeneration and tissue repair, and, to a certain extent, reducing nerve cell apoptosis.^99^ Microglia regulate their own polarization through autocrine and paracrine signaling. When infections or damage is removed, this protective reaction is downregulated. Subsequently, the M2 type gradually transforms into the M1 type, and the location of microglial cells also expands from the ischemic center to the penumbra, especially in the peri‐infarction area adjacent to ischemic neurons. The M1 phenotype markers began to increase for 14 days after 3 days of ischemia, with M1 types predominating on the 7th day and lasting for several weeks.^100^ During this phase, microglia promote the release of inflammatory factors, exacerbating brain injury and hindering regeneration and repair.^101^ All of these indicated that the polarization phenotypes of microglia under different pathophysiological conditions exert a dual role in ischemic stroke.^102^

#### 4.3.2 Phagocytic function of microglia

The large amount of cell debris produced by cell death after cerebral ischemic injury is an important factor for neuroinflammation.^103^ Microglia, especially M2 microglia, can remove cell debris by phagocytosis after an ischemic stroke, thereby reducing inflammation.^55^ In the early stage after cerebral ischemic injury, microglial phagocytosis is vital for the clearance of cell debris, whose inhibition can exacerbate neuronal injury otherwise. Studies have shown that within 3 days after middle cerebral artery occlusion in mice, which is the early stage of ischemic injury, treatment with the P2Y6 receptor inhibitor MRS2578 to inhibit microglial phagocytosis increases brain atrophy and brain edema volume. At the same time, neurological damage was aggravated.^104^

After the occurrence of ischemic stroke, microglia accumulate in and around the ischemic core area, engulfing damaged neurons, which is beneficial to reduce the inflammatory response after stroke and promote functional recovery to a certain extent.^105^ However, neurons that are still alive after an ischemia damage also release Find‐me signals, and Eat‐me signals appear on the surface, leading to phagocytosis by microglia, resulting in unnecessary neuronal loss.^106^ The role of microglia in synaptic phagocytosis has been extensively studied during development and in disease models such as Alzheimer's disease, multiple sclerosis, and schizophrenia. Evidence suggests that the role of microglia in synaptic phagocytosis is involved in synaptic pruning during development, which is also a key factor for synaptic loss in the disease models mentioned above.^107^, ^108^ After an ischemic stroke, significant synaptic loss occurs in the infarct core and penumbra, not only due to neuronal death but also because of reduced dendritic spine density in surviving neurons.^109^ Moreover, the electrophysiology of neurons in the peri‐infarct area was abnormal after an ischemic injury, indicating that the neuronal network connectivity and the plasticity of synapses in the peri‐infarct area were impaired.^110^

## 5 CONCLUSION

In summary, NVU represents a complex system where the damage and functional repair of each component are interconnected processes. The various cell types within the NVU interact with each other to form a highly integrated network, and these interconnections play a crucial role in the pathology and recovery of ischemia stroke. Recognizing and understanding these intricate connections are essential in stroke research, as they provide valuable insights into potential treatment strategies for repairing ischemic stroke injury, preserving the penumbra, and minimizing adverse outcomes.

## AUTHOR CONTRIBUTIONS

Yu Yang and Hao Tong contributed equally to this article; Yu Yang, Hao Tong, and Zhuo‐Fan Ye designed and wrote the manuscript; and Zu‐Cai Xu and Tao Tao helped with proofreading and revision. All authors have read and agreed to the published version of the manuscript.

## CONFLICT OF INTEREST STATEMENT

Zu‐Cai Xu is the associate editor of *Ibrain* and a coauthor of this article; he has not been involved in the peer review process. All decisions are left to the editor‐in‐chief so as to minimize bias. Other authors declare no conflict of interest.

## ETHICS STATEMENT

The ethics statement is not available.

## Data Availability

The data that support the findings of this study are available from the corresponding author upon reasonable request.
